# Genomic insights into population structure, resistome, virulome, and mobilome of multidrug-resistant *Pseudomonas aeruginosa*

**DOI:** 10.3389/fmicb.2026.1940877

**Published:** 2026-10-22

**Authors:** Sara Pearl, Elizabeth Annie George, Sudha Ramaiah, Anand Anbarasu

**Affiliations:** 1 Department of Biotechnology, School of Bio Sciences and Technology (SBST), Vellore Institute of Technology (VIT), Vellore, India; 2 Department of Bio-Sciences, School of Bio Sciences and Technology (SBST), Vellore Institute of Technology (VIT), Vellore, India

**Keywords:** antimicrobial resistance, Gram-negative bacteria, integrons, pangenome, *Pseudomonas aeruginosa*

## Abstract

**Background:**

*Pseudomonas aeruginosa* is a leading opportunistic Gram-negative pathogen causing severe healthcare-associated infections. The aim of the present study was to assess the interplay between population structure, antimicrobial resistance (AMR) determinants, virulence factors (VFs), and mobile genetic elements (MGEs) of *P. aeruginosa* genomes retrieved from public databases.

**Methods:**

We performed genomic profiling of 301 human-associated MDR *P. aeruginosa* genomes from clinical sources. The comprehensive genomic analyses primarily focused on characterization of sequence types (STs), antimicrobial resistance genes (ARGs), VFs, plasmids, insertion sequences (ISs), integrons, prophages, and pangenome using established bioinformatics tools.

**Results:**

The dataset exhibited diverse population structure dominated by internationally recognized high-risk STs, particularly ST621, ST277, and ST235 among the MDR genomes. The MDR *P. aeruginosa* genomes contained a greater ARG burden (mostly acquired aminoglycoside-modifying enzymes, β-lactamase genes, sulfonamide resistance genes, and disinfectant resistance determinants). Additionally, higher numbers of ISs, complete class 1 integrons, plasmid contigs, and prophage sequences, were identified supporting the pivotal role of MGEs in genome evolution and AMR dissemination. Pangenome analysis revealed an extensive accessory genome, highlighting substantial genomic diversity. In contrast, the intrinsic resistome and virulome remained highly conserved.

**Conclusion:**

MDR genomes exhibited greater burden of acquired ARGs against a conserved intrinsic resistance background. The integrated genomic framework provides insights into genomic characteristics associated with MDR *P. aeruginosa* and supports genomic surveillance of emerging MDR lineages and their associated mobile resistance elements.

## 1 Introduction

*Pseudomonas aeruginosa* (*P. aeruginosa*) is a motile, non-spore forming, non-capsulated, aerobic, Gram-negative bacilli. It is ubiquitous in nature and is resilient in various environments, often producing blue-green pigments like pyocyanin and pyoverdin. It is one among the most clinically important opportunistic pathogens responsible for healthcare-associated infections, including pneumonia, bloodstream infections, urinary tract infections (UTIs), and chronic burn wound infections. It causes severe infections in patients with cystic fibrosis, immunocompromised conditions, and indwelling medical devices and implants, especially in cases of ventilator-associated pneumonia and catheter-associated UTI. It is also associated with ear infections known as otitis externa (Diggle and Whiteley, 2020).

*Pseudomonas aeruginosa* possesses a relatively large genome, ranging from 5.5 to 7 Mbp, with a high GC content (65%–67%) and is characterized by a highly conserved core set of genes alongside an expanding and adaptive accessory genome. This genome content architecture provides striking metabolic versatility and augments adaptation through horizontal gene transfer (HGT), aiding in emergence of diverse virulence and resistance traits (Klockgether et al., 2011). A previous comparative genomics-based study revealed extensive variability among clinical *P. aeruginosa* isolates, particularly within the accessory genome, where insertion sequence (IS) elements, genomic islands (GIs), and prophages contribute to antibiotic resistance gene content variations and increased virulence repertoires (Subedi et al., 2018). The diversity in metabolic functional features, intrinsic resistance potential to multiple antibiotics, and remarkable ability to acquire additional resistance determinants have made *P. aeruginosa* one of the most difficult-to-treat bacterial pathogens. Due to its persistent stance as a leading cause of antimicrobial resistance (AMR)-associated morbidity and mortality worldwide, *P. aeruginosa* has been categorized as a “high” priority pathogen by the World Health Organization (World Health Organization, 2024). Moreover, *P. aeruginosa* is listed among the ESKAPE (*Enterococcus faecium*, *Staphylococcus aureus*, *Klebsiella pneumoniae*, *Acinetobacter baumannii*, *P. aeruginosa*, and *Enterobacter* spp.) pathogens causing multidrug-resistant (MDR) infections in nosocomial settings (Miller and Arias, 2024).

In contrast to most bacterial pathogens that depend largely on HGT of acquired resistance determinants, *P. aeruginosa* harbors an extensive intrinsic resistome comprising chromosomally encoded efflux pump systems, reduced outer membrane permeability, and inducible β-lactamases that collectively lessen antimicrobial susceptibility (Miryala et al., 2019). Additionally, acquisition of extended-spectrum β-lactamases (ESBLs), carbapenemases, aminoglycoside-modifying enzymes (AMEs), fluoroquinolone resistance genes, and disinfectant resistance determinants further contributes to MDR, extensively drug-resistant (XDR), and occasionally pandrug-resistant (PDR) phenotypes (Diggle and Whiteley, 2020). The dynamic interplay between intrinsic resistance mechanisms and acquired genes via HGT exacerbates antibacterial therapy and elevates dissemination of high-risk clones at global level. Consequent treatment failure depletes standard regimens, leaving physicians with dwindling therapeutic schemes (Kulandaivelu et al., 2026). Beyond AMR, the efficient pathogenicity of *P. aeruginosa* is mainly driven by a vast repertoire of virulence-associated genes, including multiple secretion systems, motility-associated structures, toxins, exopolysaccharide biosynthesis pathways, quorum sensing networks, proteases, siderophore systems, and adherence factors (Vadakkan et al., 2020; Marimuthu et al., 2026; Venkatesan et al., 2025). The extensive genomic plasticity of *P. aeruginosa* is further amplified by its various mobilome components, including plasmids, IS elements, integrons, GIs, and prophages. Understanding the genomic landscape underlying MDR in *P. aeruginosa* has become of escalating priority for aiding diagnostics at molecular level, enhancing surveillance programs, identifying emerging high-risk clones, and guiding antimicrobial stewardship (Basu et al., 2024; Joshi et al., 2024; Kacha and Anbarasu, 2026).

Although several whole genome sequencing (WGS)-based studies have investigated individual aspects of *P. aeruginosa* biology, comprehensive analyses simultaneously integrating resistance genotype-phenotype patterns, virulence gene profiles, and mobilome composition across diverse clinical isolates remain relatively limited. In the present study, we performed a comprehensive genomic analysis of 301 human-associated MDR *P. aeruginosa* genomes. We examined the distribution of population structure, pangenome architecture, intrinsic and acquired resistome, virulome, and mobilome landscape, across resistance phenotypes to better understand the genomic features associated with multidrug resistance and adaptation in this globally important pathogen.

## 2 Materials and methods

### 2.1 Genomic data collection

The WGS were retrieved from the NCBI Pathogen Detection database (accessed 18 February 2026). The target organism (*P. aeruginosa*) was queried in the Isolates Browser and the associated metadata was retrieved. Subsequent filtering of the data was performed based on specific inclusion criteria such as taxonomic classification, host, collection time, and the availability of the antimicrobial susceptibility testing (AST) information. This resulting curated genome assembly dataset was retrieved using NCBI Datasets Command line interface (CLI) v18.17.1 and further investigated for the downstream genomic analysis (O’Leary et al., 2024).

### 2.2 Genome assembly quality assessment

The genome quality was assessed using multiple approaches to ensure the retention of high-quality assemblies. The genome assembly metrics including the genome size, GC content, total number of contigs, N50, L50 values were assessed using QUAST v5.3.0 to assess the integrity and fragmentation of the genomes (Gurevich et al., 2013). The completeness of the genomes as represented by the presence of conserved single copy orthologs were evaluated using BUSCO v6.0.0. tool, classifying assemblies based on proportion of complete, duplicated, and fragmented gene orthologs (Manni et al., 2021a,b). In addition to this, the lineage specific completeness and contamination was evaluated using CheckM v1.1.0 (Parks et al., 2015). Whole-genome Average Nucleotide Identity (ANI) was calculated using FastANI v1.34 by aligning the query sequences with the reference genome “*Pseudomonas aeruginosa* PAO1, complete genome” (NCBI RefSeq ID: NC_002516.2), to ensure the accuracy and exclusion of contaminated genomes (Jain et al., 2018). Species-level taxonomic consistency was assessed by PathogenWatch v23.5.2 using in-built Speciator tool (Gladstone et al., 2020).

### 2.3 Antimicrobial resistance phenotype assignment

The associated AST data of the study genomes were curated to classify resistance phenotypes. Antibacterial agents to which *P. aeruginosa* is intrinsically non-susceptible, as well as agents that are no longer recommended for routine susceptibility testing according to contemporary clinical guidelines, were excluded from consideration for further phenotype assignment (Cosentino et al., 2023). The final dataset comprised clinically relevant antimicrobial agents representing eight categories defined according to the international standardized definitions of Magiorakos et al. (2012): aminoglycosides (amikacin, gentamicin, tobramycin), fluoroquinolones (ciprofloxacin, levofloxacin), monobactams (aztreonam), cephalosporins (cefepime, ceftazidime), carbapenems (imipenem, meropenem), phosphonic acid derivatives (fosfomycin), β-lactam/β-lactamase inhibitor combinations (piperacillin–tazobactam, ticarcillin–clavulanic acid), and polymyxins (colistin, polymyxin B) (Kadri et al., 2018, 2019). For each genome, individual AST results were converted into category-level interpretations. An antimicrobial category was classified as non-susceptible (NS) when at least one tested agent within that category was reported as intermediate or resistant. A category was classified as susceptible (S) when no intermediate or resistant result was reported and at least one tested agent was reported as susceptible. Categories for which AST results were unavailable for all included agents were designated as not available (NA). Resistance phenotypes were subsequently assigned according to the international standardized definitions (Magiorakos et al., 2012).

Isolates were classified as MDR when they exhibited non-susceptibility in at least three antimicrobial categories. XDR and PDR classifications were assigned only when AST information was available for all eight antimicrobial categories (NA = 0). Isolates non-susceptible to all eight categories were classified as PDR, whereas isolates non-susceptible to six or seven categories with complete category-level AST information were classified as XDR. Isolates were classified as non-MDR only when fewer than three categories were non-susceptible and the combined number of NS and NA categories was fewer than three, thereby ensuring that MDR status could be confidently excluded. All remaining isolates with incomplete AST coverage were classified as indeterminate. Only genomes classified as MDR were subsequently included for downstream genomic analyses.

The available metadata of the final MDR *P. aeruginosa* genome dataset were examined to determine their reported geographical locations and years of collection. To provide an integrated overview of this metadata in relation to genomic diversity, the collection locations and years were mapped using Microreact.

### 2.4 *In silico* typing

Using the PubMLST scheme implemented in the PubMLST *P. aeruginosa* database (Jolley et al., 2018)^1^, the sequence types (STs) of the *P. aeruginosa* isolates were predicted *in silico* directly from the WGS assemblies. Allelic profiles were determined based on the seven housekeeping genes (acsA, aroE, guaA, mutL, nuoD, ppsA, and trpE), and STs were assigned accordingly using PathogenWatch v23.5.2 (Curran et al., 2004). A minimum spanning tree (MST) illustrating the genetic relationships among identified STs was generated using GrapeTree v1.5.0 employing the MSTree algorithm with the eBURST clustering approach (Zhou et al., 2018).

### 2.5 Structural annotation and functional classification

Genome annotation was performed using Prokka v1.15.6 to predict structural gene features including coding DNA sequences (CDSs), transfer RNAs (tRNAs), ribosomal RNAs (rRNAs), transfer-messenger RNA (tmRNA), miscellaneous RNA (miscRNA), and repeat regions from assembled genomes (Seemann, 2014; Chandy et al., 2024). Functional categorization of predicted CDS was assigned using COGclassifier v2.0.0, classifying genes into Clusters of Orthologous Groups (COG) functional categories (Pearl et al., 2025).

### 2.6 Functional annotation of resistome and virulome

Antimicrobial resistance genes (ARGs) and virulence factors (VFs) were identified using ABRicate v1.2.0, with resistant determinants queried against the Comprehensive Antibiotic Resistance Database (CARD) and virulence genes detected using the Virulence Factor Database (VFDB) (Chen et al., 2016; Jia et al., 2017; Liu et al., 2022; Alcock et al., 2023). A minimum identity and coverage threshold of 80% were used to predict the genes for downstream analyses. ARGs were further manually curated and classified into intrinsic chromosomal and acquired genes based on published literature and known resistance mechanisms in *P. aeruginosa* (Murray et al., 2015). β-lactamases encoding genes were classified based on the Beta-lactamase database (BLDB) (Naas et al., 2017).

### 2.7 Functional annotation of mobilome

Insertion sequence elements were predicted using ISEScan v1.7.3, and identified ISs were categorized according to IS family (Xie and Tang, 2017). Integrons and associated gene cassette structures, including complete integrons, CALIN (Clusters of *attC* sites Lacking Integron Integrase), and In0 elements, were characterized using IntegronFinder v2.0.6 (Néron et al., 2022). Plasmid contig reconstruction and typing, including replicon types, relaxase families, mating pair formation (MPF) systems, *oriT* regions, and plasmid mobility prediction were performed using MOB-suite (MOB-recon) v3.1.9 (Robertson and Nash, 2018; Robertson et al., 2020). Prophage sequences were identified using Phigaro v2.4.0 in ‘basic’ mode, which enabled reporting of their corresponding taxonomic lineages and phage virus orthologous groups (pVOGs) (Starikova et al., 2020).

### 2.8 Genomic context and resistance gene-mobile genetic element association analysis

The genomic contexts of the major, frequently occurring acquired ARGs identified through CARD-based ABRicate screening were investigated using Flankophile v0.2.10 (Thorn et al., 2024). For each selected ARG, the genomic regions extending 1,500 bp upstream and 1,500 bp downstream of the ARG locus were extracted and examined to identify neighboring genes and mobile genetic element-associated features. The resulting flanking regions were compared across the analyzed *P. aeruginosa* genomes to identify recurrent or conserved genetic arrangements surrounding predominant acquired ARGs. This analysis enabled characterization of the local genomic environment of frequently detected resistance determinants and their potential association with surrounding mobile genetic elements.

To investigate the association of ARGs with MGEs, an integron–ARG matrix was generated by integrating the ARG and integron prediction results from IntegronFinder. ARGs identified within complete integron-associated regions were examined to determine the occurrence of ARG cassettes and their association with predicted integron structures. For plasmid-associated ARGs, predicted contigs were extracted from the plasmid reconstructions generated using MOB-suite. The predicted plasmid contigs were subjected to ARG screening using ABRicate with the CARD database. The identified ARGs were subsequently compared with the predicted plasmid mobility classifications to characterize the distribution of ARGs among conjugative, mobilizable, and non-mobilizable plasmid contigs.

### 2.9 Pangenome analysis

General Feature Format (GFF) files generated from Prokka genome annotation were used for pangenome analysis with Panaroo v1.8.0 using strict cleaning mode to minimize assembly artifacts and annotation inconsistencies (Tonkin-Hill et al., 2020). Core gene alignment was generated using MAFFT. The pangenome was classified into core, soft-core, shell, and cloud gene fractions using a core-genome sample threshold of 0.98. Genes present in ≥99% of the analyzed genomes were considered core genes, those present in 95%–<99% were classified as soft-core genes, those present in 15%–<95% as shell genes, and those present in <15% as cloud genes. Core genome-based phylogeny was reconstructed using IQ-TREE v3.1.3 with General Time Reversible (GTR) model, from the concatenated core gene alignment generated by Panaroo (Nguyen et al., 2015). The gene presence–absence matrix and core genome phylogeny was utilized to visualize the pangenome partitions and gene presence-annotated phylogenetic tree using Python scripts from Roary (Page et al., 2015).

## 3 Results

### 3.1 Genomic dataset retrieval and quality assessment

A total of *P. aeruginosa* 57,291 genome entries were available in the database from which 56,458 were retained based on organism group. Further filtering based on scientific nomenclature refined the dataset to a total of 56,451 genomes. Subsequent filtering based on the collection date from 2001 to present resulted in 43,470 isolates whereas the filtering based on host restricting isolates derived from Homo sapiens yielded 35,515 genomes. Additionally, the shortlisting based on the availability of AST phenotype data resulted in 759 genomes, out of which 643 were genome assemblies which was retained for the downstream assessments whereas the remaining 116 Sequence Read Archive (SRA) datasets were excluded for the analytical consistency. The final curated dataset of 643 assemblies with phenotypic data was further subjected for genome quality assessments.

The genome size ranged from 6.0 to 7.0 Mbp with a GC content of 65%–67%. Out of the metadata-based curated set of 643 genomes, the filtering based on number of contigs ≤200 resulted in a total of 572 genomes whereas the 568 genomes exhibited Ns per 100 kbp ≤100 indicating the high-quality assemblies. All the 568 genomes showed BUSCO completeness threshold ≥ 98.3% and CheckM completeness percentage of ≥99.94%. The contamination filtering resulted in 545 genomes with ≤2% contamination levels and the ANI analysis with ≥95% identity retained 544 genomes (Supplementary Table 1).

### 3.2 Antibiotic resistance phenotype profile and population structure

Among the final curated dataset of 544 *P. aeruginosa* genomes, 301 (55.3%) were classified as MDR, 15 (2.8%) as non-MDR, and 228 (41.9%) as indeterminate based on the available AST data. No XDR or PDR phenotypes could be confidently assigned under the applied completeness criteria because phosphonic acid (fosfomycin) AST data were unavailable for all genomes, resulting in incomplete eight-category AST profiles. Only MDR genomes were subsequently included for downstream genomic analyses (Supplementary Table 2). The 301 MDR genomes represented 8 countries and were collected between 2004 and 2023 (Supplementary Figure 1).

MLST analysis identified 62 different STs among the 298 MDR genomes with assigned sequence types, demonstrating substantial population diversity. Nevertheless, the dataset was strongly dominated by ST621, accounting for 198 genomes (65.8%), followed by ST277 (*n* = 23, 7.6%), ST235 (*n* = 14, 4.7%), and ST973 (*n* = 3, 1.0%). Three genomes had no assigned ST, while the remaining STs were represented by two or fewer genomes each (Supplementary Table 3). The minimum spanning tree demonstrated the predominance of MDR genomes within the ST621 lineage, with additional clustering observed among ST277 and ST235 (Figure 1).

**FIGURE 1:**
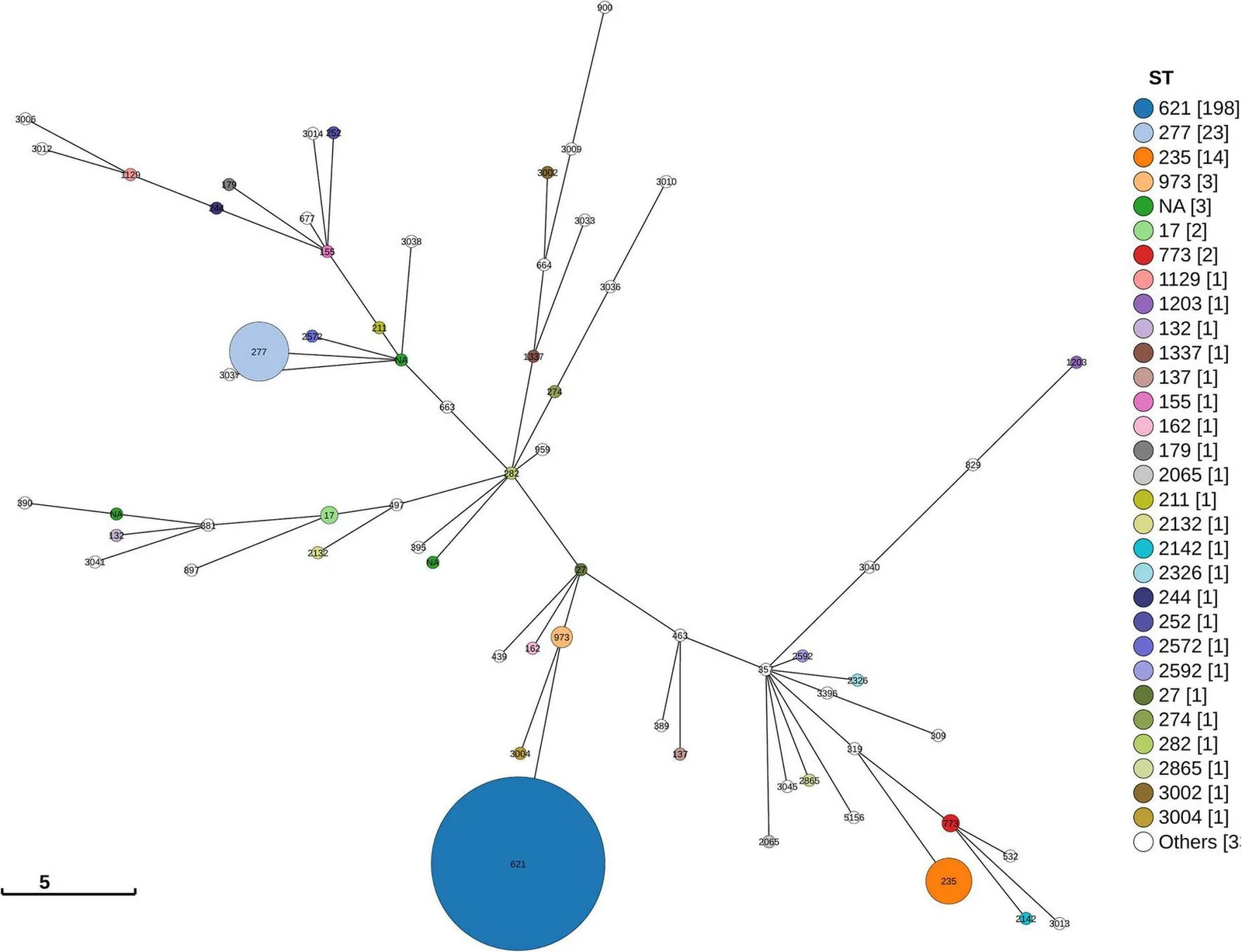
Minimum spanning tree (MST) depicting the population structure and sequence type (ST) distribution of 301 *P. aeruginosa* clinical genomes.

### 3.3 Structural genomic architecture

Genome annotation identified an average of 6,460 genes per genome, including 6,271 CDSs, 121 miscRNAs, 63 tRNAs, 3 rRNAs, 3 repeat regions, and a single tmRNA on average (Supplementary Table 4). These findings are consistent with the complex genomic organization of *P. aeruginosa*, indicating its large coding capacity and extensive regulatory repertoire. Functional annotation of the predicted CDSs using COGclassifier revealed that genes associated with the mobilome: prophages and transposons (COG X), had an average abundance of 74 genes per genome among the MDR genomes, ranging from 21 to 122 genes per genome (Supplementary Table 5). This suggests a greater burden of mobile genetic elements (MGEs) among MDR *P. aeruginosa* population, consistent with their enhanced capacity for horizontal gene acquisition and genome plasticity.

### 3.4 Resistome profile

A total of 16,705 ARGs representing 167 unique genes were identified across the 301 MDR genomes, with an overall mean of 56 ARGs per genome, ranging from 49 to 72. The resistome of the MDR population comprised both conserved intrinsic resistance determinants and diverse acquired ARGs associated with multiple antimicrobial classes.

#### 3.4.1 Intrinsic resistome

The intrinsic resistome encompassed multiple antimicrobial classes, particularly phenicol, tetracycline, macrolide, disinfecting agents and antiseptics, fluoroquinolone, aminoglycoside, β-lactams, and aminocoumarins. The intrinsic resistome comprised 84 gene entries, with a total of 12,328 gene detections across the 301 MDR genomes. The most prevalent intrinsic determinants were *aph*(3′)-IIb, *arnA*, and *fosA*, each detected in all 301 genomes. The intrinsic efflux-associated repertoire was dominated by the genes encoding Mex and Opr systems, including MexA, MexB, MexC, MexD, MexE, MexF, MexG, MexH, MexI, MexJ, MexK, MexV, MexW, MexX, MexY, OpmB, OpmD, OpmH, OprJ, OprM, and OprN, which were detected in 299–301 genomes.

The intrinsic β-lactamase repertoire was dominated by the genes encoding PDC and OXA families. The PDC family comprised 15 variants, including classical/wild-type, extended-spectrum class C β-lactamase (ESAC), and inhibitor-resistant classifications. PDC-374 was the predominant PDC determinant, detected in 283 genomes (94.0%). The intrinsic OXA repertoire was primarily composed of genes coding for OXA-5-like and OXA-1-like determinants. OXA-914 was the most prevalent intrinsic OXA determinant, detected in 198 genomes (65.8%), followed by OXA-494 (*n* = 28; 9.3%) and OXA-488 (*n* = 17; 5.6%). The remaining OXA-5-like variants were detected sporadically, indicating substantial allelic diversity within the intrinsic β-lactamase repertoire.

Overall, these observations suggest that the intrinsic resistome of *P. aeruginosa* is characterized by a highly conserved chromosomal ARG repertoire.

#### 3.4.2 Acquired resistome

The acquired resistome was dominated by aminoglycoside resistance determinants, followed by bicyclomycin-like, disinfecting agents and antiseptics, sulfonamide, and β-lactam resistance. Additional acquired determinants were associated with phenicol, carbapenem, diaminopyrimidine, macrolide, tetracycline, monobactam, fluoroquinolone, streptogramin, and rifamycin resistance. The acquired resistome comprised 73 acquired gene entries, with 1,673 gene detections across the 301 genomes. The most prevalent acquired determinants were bcr-1 (*n* = 299), sul1 (*n* = 248), qacEΔ1 (*n* = 248), aac(6′)-Ib7 (*n* = 223), and aadA (*n* = 202).

The acquired β-lactamase repertoire comprised diverse class A, class B, and class D determinants. Gene encoding OXA-2 was the predominant acquired OXA determinant, detected in 200 genomes (66.4%), followed by OXA-56 (*n* = 23; 7.6%). The acquired class A repertoire included genes coding for GES, KPC, PAU, PME, TEM, and VEB determinants, while the class B repertoire included HMB-2, IMP, NDM, and VIM variants. The class D repertoire included SPM-1, detected in 20 genomes (6.6%). Several acquired carbapenemases, including GES-20, IMP-16, IMP-45, KPC-2, NDM-1, SPM-1, and VIM-80, were detected at low frequencies (Figure 2).

**FIGURE 2:**
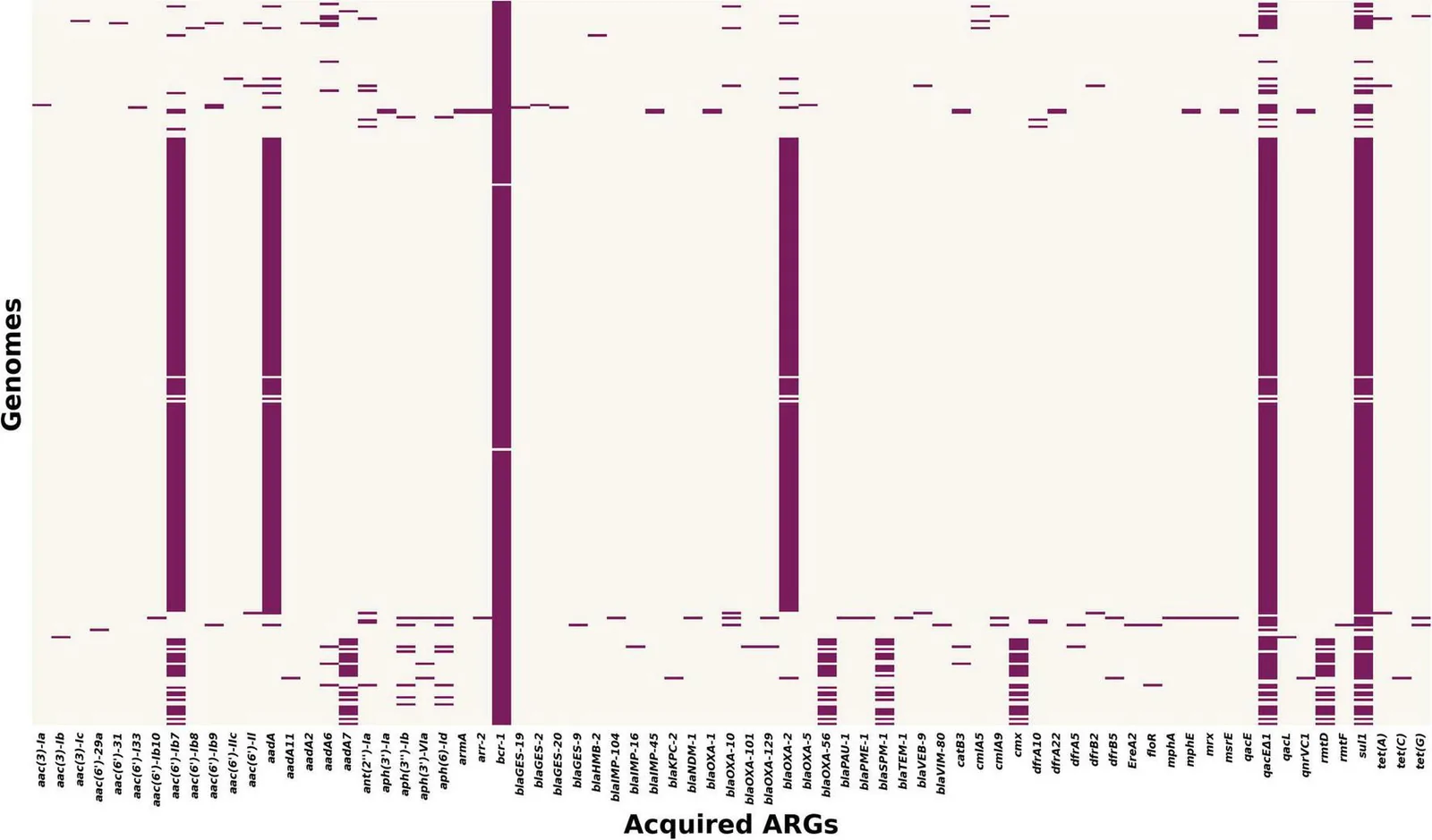
Heatmap showing distribution of 73 acquired antimicrobial resistance genes (ARGs) among *P. aeruginosa* genomes, annotated with antibiotic resistance phenotype category.

Collectively, the acquired resistome was characterized by a prevalence of aminoglycoside resistance genes and integron-associated ARGs, whereas clinically important acquired carbapenemases remained relatively infrequent.

#### 3.4.3 Regulatory elements

In addition to the intrinsic and acquired resistomes, the dataset contained nine regulatory-element entries, including armR, basS, cprR, cprS, parR, parS, cpxR, soxR, and rsmA, with occurrence ranging from 299 to 301 genomes.

#### 3.4.4 Lineage-associated distribution of resistance genes

Population structure-based distribution of intrinsic resistance determinants revealed aph(3′)-IIb, arnA, fosA, multiple components of the mexAB-oprM, mexCD-oprJ, mexEF-oprN, mexGHI-opmD, mexJK, muxABC, and triABC efflux systems, as well as pmpM, emrE, and yajC, showed identical or near-identical distributions between ST621 and non-ST621 genomes. Among intrinsic β-lactam resistance determinants, the distribution of genes encoding PDC and OXA variants showed greater lineage-associated variation. bla_*PDC–374*_ was identified in 283 genomes, including all 198 ST621 genomes and 85 non-ST621 genomes. In contrast, bla_*OXA–914*_ was detected exclusively in ST621, occurring in all 198 ST621 genomes and absent from the 103 non-ST621 genomes. Several other genes coding for intrinsic OXA variants, including OXA-488 (*n* = 17), OXA-494 (*n* = 28), OXA-50 (*n* = 6), and OXA-395 (*n* = 7), were detected exclusively among non-ST621 genomes. Multiple additional OXA and PDC variants occurred at low frequencies and were restricted to the non-ST621 group.

The distribution of the predominant acquired ARGs showed marked differences between ST621 and non-ST621 genomes. Among ST621 genomes (*n* = 198), *aac*(6′)-Ib7, *aadA*, *bla*_*OXA–2*_, *qacE*Δ*1*, and *sul1* were each detected in 195 genomes (98.5%). In comparison, *aac*(6′)-Ib7 (*n* = 28; 27.2%), *aadA* (*n* = 7; 6.8%), *bla*_*OXA–2*_ (*n* = 5; 4.9%), and *qacE*Δ*1*(*n* = 53; 51.5%) showed varied distribution among non-ST621 lineages (*n* = 103). bcr-1 was detected in 196 (99.0%) ST621 genomes and all 103 non-ST621 genomes. Several acquired ARGs were detected exclusively among non-ST621 genomes, including *aadA7* (24/103; 23.3%), *rmtD* (23/103; 22.3%), *bla*_*SPM–1*_ (20/103; 19.4%), *cmx* (25/103; 24.3%), and *bla*_*OXA–56*_ (23/103; 22.3%). Additional acquired determinants, including genes encoding GES, IMP, KPC-2, NDM-1, VEB-9, and VIM-80 variants, were detected at low frequencies and were restricted to non-ST621 genomes.

Overall, the marked differences in the distribution of several predominant ARGs between ST621 and non-ST621 genomes indicate substantial variation in resistome composition within the MDR population.

### 3.5 Virulome profile

Virulence factor screening detected virulence determinants associated with adherence, biofilm formation, effector delivery systems, exotoxin production, immune modulation, motility, and nutrient acquisition across the studied 301 *P. aeruginosa* genomes. The majority of VFs were highly conserved, suggesting the presence of a large core virulence gene repertoire irrespective of AMR phenotype.

#### 3.5.1 Colonization, adherence and biofilm-associated determinants

The adherence-associated repertoire was dominated by Type IV pili and Fap systems. Among Type IV pili-associated genes, chpB, chpC, chpD, chpE, pilF, pilG, pilH, pilI, pilJ, pilK, pilM, pilO, pilP, pilQ, pilT, and vfr were detected in all 301 genomes. Other highly prevalent determinants included fimL, fimX, pilB, and rpoN/rpoS, which were detected in 288–300 genomes. The Fap repertoire was also highly prevalent, with fapA, fapB, fapD, fapE, and fapF present in all genomes, while fapC was detected in 266 genomes. The biofilm-associated repertoire comprised alginate biosynthesis, quorum-sensing, and regulatory determinants. Most alginate-associated genes, including algA, algB, algD, algE, algF, algG, algI, algJ, algK, algL, algQ, algR, algU, algZ, alg8, alg44, and mucA–mucE, were detected in 299–301 genomes. The algP/algR3 locus was detected in 162 genomes. Quorum-sensing genes lasI and lasR were present in all genomes, while rhlI and rhlR were detected in 299 genomes.

#### 3.5.2 Motility, secretion and host-interaction factors

The motility-associated repertoire was dominated by flagellar genes, most of which were detected in nearly all genomes. The majority of flg, fli, flh, mot, fle, and che genes were present in 299–301 genomes. Other motility-associated genes, including PA1458, PA1459, PA1464, PA3348, and PA3349, were detected in 293–301 genomes.

The effector delivery repertoire was characterized by the presence of Type III secretion system (T3SS), Type VI secretion system (T6SS), HSI-1/HSI-2/HSI-3, and Xcp secretion system genes. Most core secretion-system components were detected in nearly all genomes. The T3SS repertoire included *exsA*–*exsE*, *pcr1*–*pcr4*, *pcrD*, *pcrG*, *pcrH*, *pcrR*, *pcrV*, *popB*, *popD*, *popN*, and *pscB*–*pscU*, with most present in 299–301 genomes. The T3SS effector genes, *exoS* was detected in 273 genomes, *exoT* in 300, *exoU* in 28, and *exoY* in 240 genomes. The HSI-1 repertoire was also highly conserved, with most structural and regulatory components detected in 299–301 genomes. The HSI-2/HSI-3 repertoire included several highly prevalent components, including *hcp*/*tssD*, *icmH*/*tssL*, *tssA*–*tssH*, *tssI*, *tssJ*, *tssK*, and *tssM*. The Xcp secretion system was highly conserved, with *xcpP*–*xcpZ* detected in 300–301 genomes. The repertoire also included the secreted effectors *lasA* and *lasB*, which were detected in 301 and 300 genomes, respectively.

The exoenzyme/exotoxin repertoire included aprA, plcH, and toxA. aprA and plcH were detected in all 301 genomes, while toxA was detected in 299 genomes.

#### 3.5.3 Immune evasion and host-adaptation determinants

The immune modulation-associated repertoire comprised LPS, rhamnolipid, and other immune-associated determinants. Several LPS-associated genes, including *waaA*, *waaC*, *waaF*, *waaG*, *waaP*, and *wbpM*, were detected in all 301 genomes. The rhamnolipid-associated genes *rhlA*, *rhlB*, and *rhlC* were detected in 300 genomes.

#### 3.5.4 Nutrient acquisition and metabolic virulence determinants

The nutritional/metabolic repertoire included pyochelin, pyoverdine, and pyocyanin biosynthesis-associated genes. The pyochelin repertoire was highly prevalent, with *fptA*, *pchA*, *pchB*, *pchC*, *pchF*, *pchG*, *pchI*, *pchR*, PA4218, PA4219, and PA4220 detected in 299–301 genomes. The pyoverdine-associated repertoire was more variable, with *fpvA* and *pvdD* detected in less frequency, while *fpvR*, *mbtH*-like, PA2383, PA2384, *pvdF*, *pvdG*, *pvdH*, *pvdO*, *pvdP*, and *pvdS* were detected in 300–301 genomes. The pyocyanin repertoire showed substantial variation among biosynthesis-associated genes. *phzM* was detected in all 301 genomes, while *phzH*, *phzS*, *phzC1*, *phzD1*, *phzE1*, *phzF1*, and *phzG2* were detected in 252–300 genomes.

### 3.6 Mobilome profile

#### 3.6.1 Insertion sequences

Insertion sequence elements were detected across all 301 *P. aeruginosa* genomes, with a total of 6,332 copies and an average of 21 copies per genome. The number of IS elements ranged from 2 to 45 copies per genome, indicating substantial variation in the abundance of MGEs among the analyzed genomes. The IS repertoire comprised of 17 recognized IS families and a small group of elements classified as “new.” The IS3 family was the most abundant, accounting for 2,958 copies (46.7%), followed by IS5 with 883 copies (13.9%), IS110 with 452 copies (7.1%), IS21 with 443 copies (7.0%), and ISL3 with 421 copies (6.6%). Together, these five families represented approximately 81.3% of all detected IS copies. Other relatively abundant families included ISNCY (268 copies; 4.2%), IS66 (220 copies; 3.5%), IS6 (217 copies; 3.4%), and IS256 (153 copies; 2.4%). The remaining families including IS481, IS1182, IS91, IS30, IS200/IS605, IS4, IS630, and IS1380, were detected at lower frequencies (Supplementary Figure 2). Overall, the IS profile was strongly dominated by the IS3 family, while several additional IS families occurred at lower copy numbers, demonstrating a diverse but variedly distributed IS repertoire across the dataset.

#### 3.6.2 Integrons

The presence of integrons was predicted in 251 of the 301 *P. aeruginosa* genomes (83.4%), with a total of 291 integrons identified across the dataset. Among the detected integrons, 245 were classified as complete, while 38 were classified as CALINs and 8 as In0 elements. Complete integrons therefore represented the predominant integron category, accounting for approximately 84.2% of all detected integrons. Among complete integrons, attC sites were the most frequently detected structural components (*n* = 672), followed by intI (*n* = 246) and attI1 (*n* = 240). These findings indicate that the identified complete integrons commonly contained the core structural components required for integron-mediated gene capture and rearrangement. Resistance-associated cassette elements were also detected within the integrons. Overall, the integron repertoire was characterized by a high prevalence of complete integrons and a predominance of attC, intI, attI1, and several recurrent antimicrobial-resistance-associated cassette elements.

#### 3.6.3 Plasmids

Plasmid-associated contigs were detected in 279 of the 301 *P. aeruginosa* genomes (92.7%), corresponding to 563 predicted plasmid contigs and an average of approximately two contigs per plasmid-positive genome. Replicon typing was successful for 209 of the 563 plasmid contigs (37.1%), whereas 354 contigs (62.9%) lacked an assigned replicon type. Among the assigned replicons, rep_cluster_322 was the most frequent type, occurring in 131 contigs, followed by rep_cluster_2238 in 69 contigs. Relaxase genes were identified in 98 plasmid contigs (17.4%), comprising MOBP in 70 contigs (12.4%) and MOBH in 28 contigs (5.0%). The remaining 465 contigs (82.6%) lacked an assigned relaxase type. Mating-pair formation (MPF) systems were detected in 191 contigs (33.9%). The most frequent system was MPF_*G*_, identified in 112 contigs (19.9%), followed by MPF_*T*_ in 68 contigs (12.1%). MPF_*I*_ and MPF_*F*_ were detected in six and five contigs, respectively. No origin of transfer (*oriT*) types were detected. Overall, 96 contigs (17.1%) were predicted to be conjugative, two (0.4%) mobilizable, and 465 (82.6%) non-mobilizable. These findings indicate a substantial plasmid-associated sequence burden in the dataset.

#### 3.6.4 Prophages

Prophages were detected in all 301 *P. aeruginosa* genomes, with a total of 1,444 prophage regions and an average of 4.80 prophages per genome. The number of prophages ranged from 1 to 8 per genome. Taxonomic classification identified *Siphoviridae* as the predominant group, accounting for 628 prophages (43.5%), followed by *Myoviridae* with 353 prophages (24.4%). Among the detected prophages, 133 were classified as transposable prophages and were identified in 129 genomes. These genomes contained an average of one transposable prophages, with a range of 1–2 per genome. Most transposable prophages had unknown taxonomy (101/133; 75.9%), followed by *Myoviridae* (23/133; 17.3%) and *Siphoviridae* (7/133; 5.3%).

### 3.7 Antimicrobial resistance gene and mobile genetic element association

#### 3.7.1 Genomic context and co-localization of major acquired ARGs

Genome-context analysis of *bcr-1* revealed a recurrent local genetic environment across the analyzed *P. aeruginosa* genomes. The *bcr-1* gene was frequently associated with *bpeR* in its upstream genomic neighborhood, with this association observed across multiple *bcr-1*-positive genomes. An *acrR*-associated upstream context was observed in a subset of genomes (Figure 3A). Genomic context of *qacE*Δ*1* exhibited a highly recurrent genetic arrangement with a predominant configuration consisting of *ant1*–*bla*–*qacE*Δ*1*–*folP* repeatedly observed across the analyzed genomes, with qacEΔ1 consistently positioned between the upstream resistance-associated genes and *folP*. Although this conserved arrangement predominated, variation in the flanking regions was also observed. Notably, IS*Pa62* was detected in the upstream region of *qacE*Δ*1* in one genome, indicating the presence of an IS-associated context. Additional resistance-associated genes, including *aacA4*, were observed in some variable genomic contexts (Figure 3B).

**FIGURE 3:**
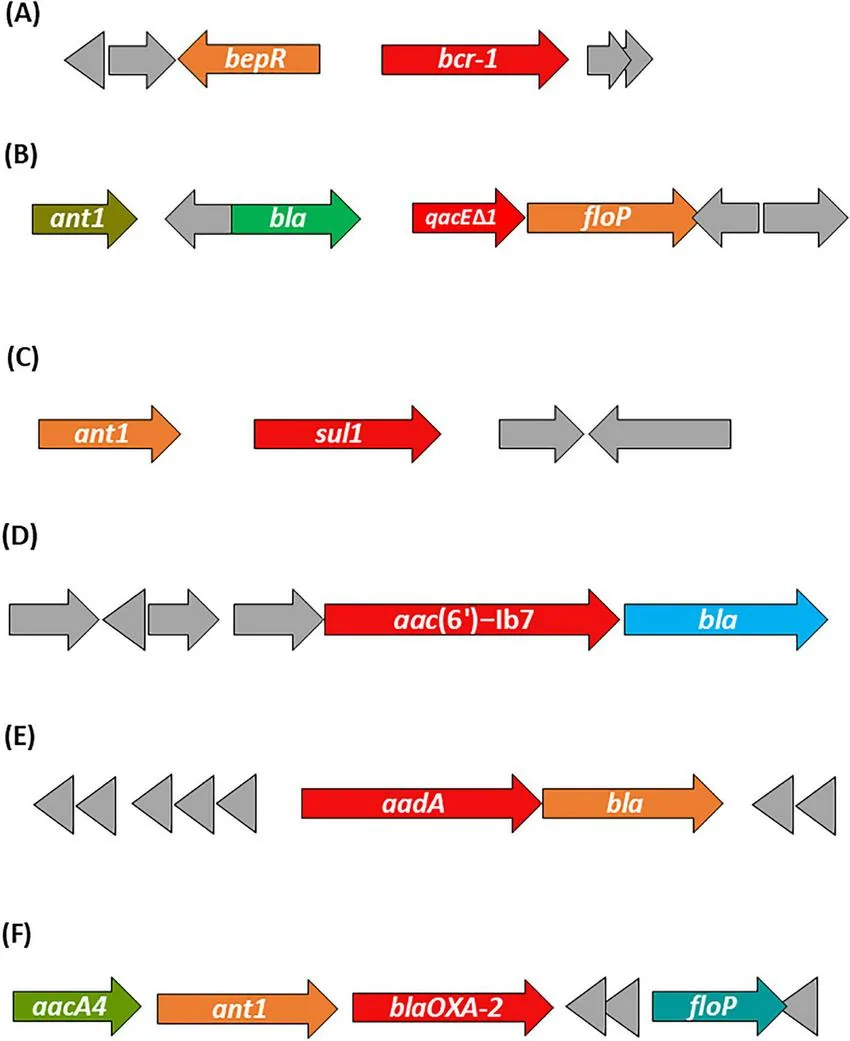
Representative genome context of predominant acquired antimicrobial resistance genes in 301 MDR *P. aeruginosa* **(A)** *bcr-1* **(B)** *qacE*Δ*1* **(C)** *sul1* **(D)** *aac*(6′)-Ib7 **(E)** *aadA* and **(F)** *bla*_*OXA–2*_. Gene neighborhoods are shown with flanking genes and mobile genetic element-associated features identified within the analyzed genomic context. The predominant genomic arrangements observed for each ARG are presented. An IS6100 element and other associated resistance genes/MGE features are shown where present in the respective contexts.

Genome context of sul1 showed a predominantly conserved genomic environment with ant1 as the most frequently observed upstream neighboring resistance-associated gene, occurring repeatedly adjacent to sul1. In addition to ant1, variable upstream contexts included other resistance-associated determinants such as aacA4, aacC1, cat, and adh. The upstream region also showed occasional association with MGE-related sequences, including IS6100. Considerable variation was observed in the downstream flanking regions, although several genomes retained similar local arrangements (Figure 3C).

The predominant arrangement showed *aac*(6′)-Ib7 immediately followed by a *bla*-associated gene, and this configuration was repeatedly observed across the analyzed genomes. In a smaller number of genomic contexts, *hin* was observed downstream of *aac*(6′)-Ib7. Variation was also observed in the upstream flanking regions, although several genomes retained similar local arrangements (Figure 3D). The *aadA* contexts similarly demonstrated strong association with β-lactam resistance-associated genes. A recurrent *aadA*–*bla* arrangement was observed across numerous genomic contexts, with the orientation of the two genes varying in some cases. Additional neighboring sequences included *folP* and *aacA4*-associated genes (Figure 3E).

Genome context of *bla*_*OXA–2*_ revealed that frequent localization within a conserved resistance-associated region containing *ant1* and *aacA4*, with *folP* commonly occurring downstream. In several instances, *bla*_*OXA–2*_ was positioned directly adjacent to *ant1*, while *aacA4* was observed upstream, indicating recurrent co-localization of aminoglycoside- and β-lactam-resistance determinants within the same genomic context. The repeated occurrence of this arrangement suggests persistence of a conserved resistance-associated genetic structure rather than random distribution of the individual ARGs (Figure 3F).

#### 3.7.2 Integron-associated resistance determinants and cassette composition

The most frequently detected resistance-associated cassette elements included *aac*(6′)-Ib (*n* = 208), *qacE* (*n* = 202), *ant*(3″)-Ia/*aadA1* (*n* = 196), and *bla*_*OXA–2*_ (*n* = 195). The frequent occurrence of aminoglycoside-resistance, antiseptic/disinfectant-resistance, and β-lactamase-associated cassette elements suggests that integrons contributed substantially to the organization and dissemination of acquired antimicrobial-resistance determinants in the analyzed genomes. Less frequent cassette-associated elements included *ant*(3″)-I (*n* = 11), *ant*(2″)-Ia (*n* = 9), *bla*_*OXA–10–like*_ (*n* = 8), and *bla*_*GES*_ (*n* = 3). Several additional resistance-associated elements, including *aac*(3)-I, *catB*, *cmlA*, *aac*(6′)-IIc, *bla*_*IMP*_, *bla*_*VIM*_, *ereA*, *qnrVC*, *arr*, and *dfrA12*/*A21*, were detected only sporadically. Collectively, the repeated co-occurrence of aminoglycoside-resistance genes, *qacE*, and *bla*_*OXA–2–like*_ suggests that class 1 integrons contributed to the accumulation of multidrug-resistance-associated cassette combinations in the dataset.

#### 3.7.3 Plasmid-associated antimicrobial-resistance genes

Among the 563 plasmid contigs predicted across the 301 MDR *P. aeruginosa* genomes, 362 (64.3%) were positive for at least one resistance determinant, encompassing 46 distinct ARGs. The most frequently detected determinants were *cpxR* (*n* = 234), *qacE*Δ*1* (*n* = 96), *sul1* (*n* = 89), *aac*(6′)-Ib7 (*n* = 83), *bla*_*OXA–2*_ (*n* = 76), and *aadA* (*n* = 74). Other frequently detected determinants included *cmx* (*n* = 23), *aadA7* (*n* = 7), *dfrA10* (*n* = 7), *bla*_*OXA–56*_ (*n* = 7), and *ant*(2″)-Ia (*n* = 6). Several acquired carbapenemase genes, including *bla*_*IMP–104*_, *bla*_*IMP–16*_, *bla*_*KPC–2*_, and *bla*_*NDM–1*_, were identified on individual plasmid contigs, while *bla*_*IMP–45*_ was detected on two contigs.

MOB-typer classification of the 362 ARG-positive plasmid contigs identified 67 (18.5%) as conjugative, one (0.3%) as mobilizable, and 294 (81.2%) as non-mobilizable. Thus, 68 ARG-positive plasmid contigs (18.8%) showed predicted mobilization potential. Among the conjugative plasmid-associated resistance determinants, the co-occurrence of *aac*(6′)-Ib7, *aadA*, *cpxR*, *qacE*Δ*1*, and *sul1* was particularly prominent, indicating that conjugative plasmids can carry combinations of resistance determinants spanning multiple antimicrobial classes and associated resistance mechanisms. Overall, a subset of ARG-positive plasmids with predicted conjugative potential were identified, highlighting their potential importance in the horizontal dissemination of multidrug resistance-associated determinants.

### 3.8 Pangenome profile

Pangenome analysis of the 301 MDR *P. aeruginosa* genomes identified a total of 14,741 gene clusters, comprising core and accessory genes. The core genome consisted of 5,124 (34.8%) strict-core genes present in ≥99% of genomes. The accessory genome comprised 113 (0.8%) soft-core genes, 1,315 (8.9%) shell genes, and 8,189 (55.6%) cloud genes (Figure 4A). Overall, the accessory genome consisted of 9,617 genes (65.2%), substantially exceeding the conserved core genome and demonstrating extensive genomic diversity within the study population. The core genome phylogeny annotated with the pangene clusters presence and absence matrix is presented in Figure 4B. The large proportion of shell and cloud genes indicates considerable variability in gene content among isolates, reflecting the influence of HGT and niche-specific adaptation.

**FIGURE 4:**
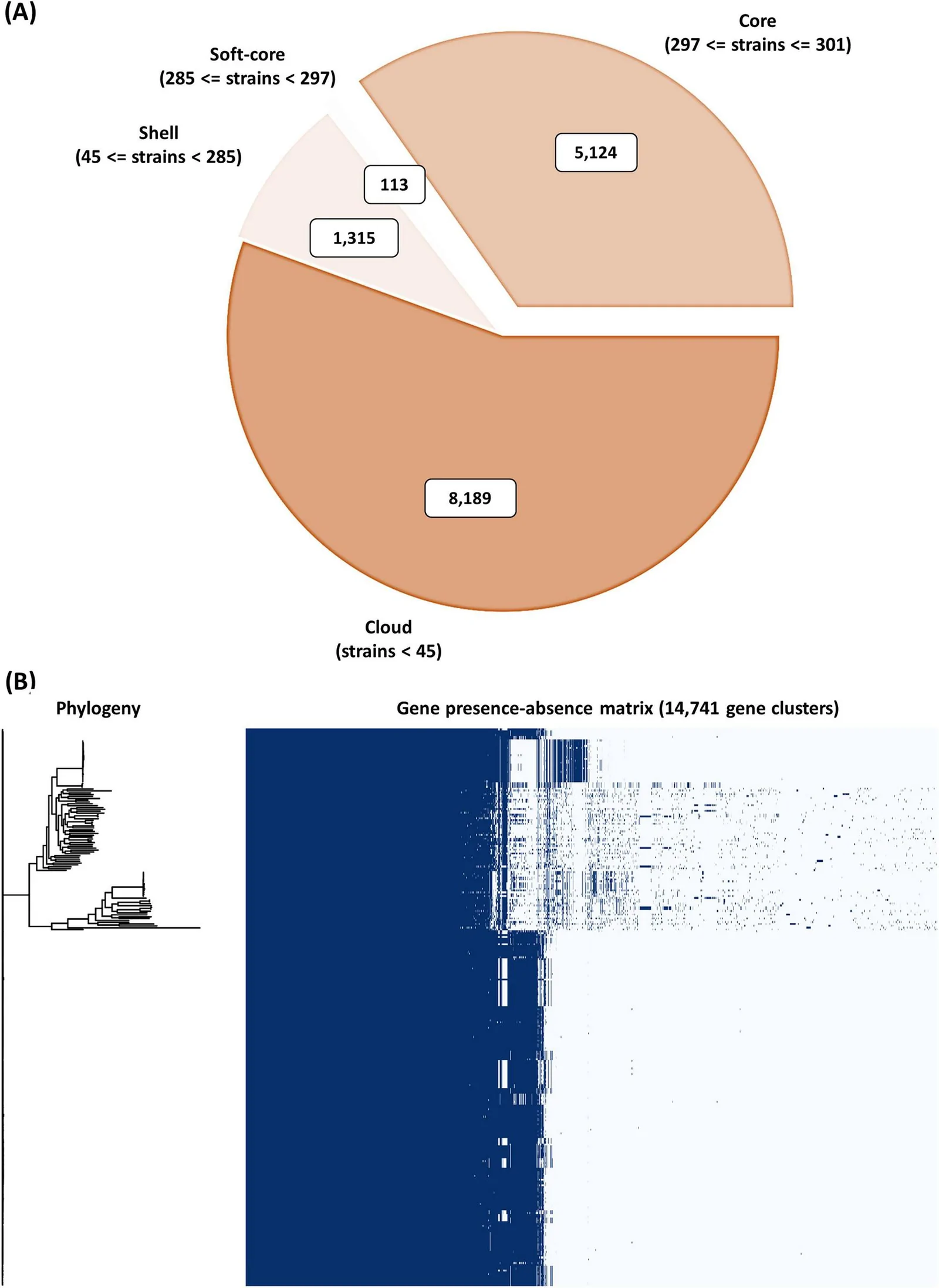
Pangenome architecture of 301 clinical *P. aeruginosa* genomes. **(A)** Partitioning of the pangenome into core, soft-core, shell, and cloud gene cluster groups **(B)** Core genome-based phylogeny with the corresponding gene presence–absence matrix illustrating the distribution of accessory genes across the genomes.

## 4 Discussion

Multidrug-resistant *P. aeruginosa* is a highly dynamic opportunistic pathogen with diverse population structure, pangenome composition, ARGs, VFs, and MGEs. The current study provides a comprehensive genomic characterization of 301 human-associated MDR *P. aeruginosa* genomes insights into the evolutionary mechanisms underlying the resistance and adaptation of this opportunistic pathogen. The population structure analysis among MDR isolates revealed the predominance of high-risk clones, including ST621, ST277, and ST235, which have previously been reported to be associated with hospital outbreaks, carbapenem resistance, and global dissemination. The prevalence of these STs underscores their clinical importance (Khuntayaporn et al., 2019; Del Barrio-Tofiño et al., 2020).

Resistome analysis showed that MDR *P. aeruginosa* harbored a higher number of resistance genes which can be largely driven by the acquisition of resistance determinants *via* HGT. The resistome profile comprised of genes encoding conserved intrinsic components which are dominated by multidrug efflux systems (Mex, Opr, and Opm), chromosomal AmpC (PDC) variants, FosA, and APH(3′)-IIb, along with a diverse repertoire of acquired resistance associated determinants. ESAC variants represented the predominant PDC subtype within the study population, whereas classical and inhibitor-resistant PDC alleles were comparatively uncommon. AMEs, β-lactamases, and class 1 integron-associated ARGs (*aadA*, *sul1*, and *qacE*Δ*1*) were the most prevalent acquired ARGs identified in the genomes. The variation within intrinsic β-lactamases were primarily characterized by genes coding for OXA-50-like and PDC allelic variants rather than differences in gene presence, consistent with their lineage-associated evolution. The predominance of acquired ARGs across the MDR genomes highlights the importance of HGT in shaping the resistance profiles. Enrichment of integron-associated ARGs, AMEs, and acquired β-lactamases revealed that MGEs continue to drive the emergence of difficult-to-treat *P. aeruginosa* infections (Zhao et al., 2023; Fernández-Vázquez et al., 2026). These findings underscore the urgent need for continuous genomic surveillance along with conventional AST in order to facilitate the early detection of high-risk MDR clones and support the infection control strategies. The virulome profile revealed a highly conserved repertoire of adherence, biofilm-associated, secretion-system, motility, and nutritional/metabolic determinants, alongside variable distribution of selected effector, immune modulation, and secondary metabolite-associated genes across the 301 MDR *P. aeruginosa* genomes.

The profiling of MGEs further underscored their contribution in genome evolution. The MDR *P. aeruginosa* genomes carried higher burden of ISs, plasmid contigs, complete integrons, and prophages, collectively indicating enhanced mobility of the genomes. IS landscape demonstrated the dominance of IS3, IS5, IS110, IS21, and ISL3 families, augmenting the potential for gene mobilization and genomic rearrangements. IS3 and IS5 are frequently associated with genome rearrangements, gene mobilization, and modulation of resistance gene expression whereas the IS6 enrichment are commonly associated to the mobilization of resistance genes (Lipszyc et al., 2022). The abundance of IS110 and ISL3 in MDR genomes may indicate the ongoing acquisition of foreign genetic material and genomic restructuring. Similarly, complete class 1 integrons frequently carried multiple ARG cassettes. Furthermore, MDR genomes not only harbor integrons more frequently but also possess more densely populated resistance cassette arrays with MDR-associated integrons revealing greater cassette complexity (Khademi et al., 2021; Yalda et al., 2021). Prophage sequences were identified in all genomes, indicating their widespread contribution to genome diversification, although transposable prophages represented only a subset of the overall prophage population (Xavier et al., 2025).

Genome context analysis demonstrated that the predominant acquired ARGs were frequently embedded within conserved and recurrent resistance-associated regions, supporting their potential persistence and co-selection in *P. aeruginosa*. The *bcr-1* context showed repeated association with a highly conserved local genomic arrangement, whereas *qacE*Δ*1* and *sul1* were consistently detected within class 1 integron-associated regions, highlighting the importance of integron structures in the accumulation and maintenance of resistance and disinfectant-resistance determinants. Notably, an IS*Pa62* element was observed upstream of *qacE*Δ*1* in one representative context, suggesting the presence of an additional MGE-associated feature surrounding this resistance region. The aminoglycoside-resistance genes *aac*(6′)-Ib7 and *aadA* also exhibited recurrent genomic associations. *aac*(6′)-Ib7 was frequently positioned adjacent to a *bla* gene, while *aadA* commonly occurred within a conserved *aadA*–*bla* arrangement, indicating co-localization of aminoglycoside- and β-lactam-resistance determinants. Similarly, *bla*_*OXA–2*_ repeatedly occurred within a conserved region containing *aacA4*, *ant1*, and downstream *folP*, further demonstrating the clustering of multiple resistance determinants within specific genomic neighborhoods. Collectively, these conserved ARG contexts suggest that resistance determinants in *P. aeruginosa* are not distributed randomly but can occur in recurring genetic configurations that may facilitate their persistence and co-selection.

Furthermore, the identification of ARGs on plasmid contigs with predicted conjugative mobility provides an important genomic indication of their potential contribution to horizontal antimicrobial resistance dissemination in *P. aeruginosa*. Although the majority of ARG-positive plasmids were classified as non-mobilizable, the detection of 67 conjugative plasmids demonstrates that a subset of resistance-bearing plasmid elements possesses predicted mobility characteristics that could facilitate transfer between bacterial cells. Conjugative plasmids are particularly relevant to AMR dissemination because their self-transmissible nature can enable the movement of linked resistance determinants across bacterial populations (Rodríguez-Beltrán et al., 2021). The ARG composition of the conjugative plasmid-associated dataset further suggests that plasmid mobility may contribute to the maintenance and dissemination of multidrug resistance determinants. The frequent occurrence of *aac*(6′)-Ib7, *aadA*, *bla*_*OXA–2*_, *sul1* and *qacE*Δ*1*, together with other aminoglycoside, β-lactam, sulfonamide and biocide-associated resistance determinants, indicates that individual mobile genetic backgrounds may harbor resistance determinants affecting multiple antimicrobial classes.

The pangenome analysis revealed a highly diverse genomic architecture characterized by highly variable accessory genome and a conserved core. This genomic plasticity reveals the adaptive potential *via* gene gain or loss events, while conserving the essential housekeeping properties. This variability in the accessory genomes is associated with the adaptability across diverse environments.

Collectively, these findings reveal the interplay between the highly conserved intrinsic resistance framework and the progressive acquisition of resistance determinants mediated by plasmids, integrons, ISs, and prophages. The conservation of major virulence factors along with increasing resistance gene burden indicates that successful MDR lineages retain their pathogenic potential while simultaneously expanding their antimicrobial resistance repertoire. The sustained conservation of core virulence determinants further indicates that successful MDR lineages maintain substantial pathogenic potential despite the acquisition of the resistance genes. Consequently, therapeutic failure in MDR *P. aeruginosa* infections likely attribute not only to antimicrobial resistance but also to the persistence of robust virulence mechanisms that promote host colonization, biofilm formation, immune evasion, and long-term persistence.

This study has several limitations as the resistance mechanisms associated with chromosomal mutations, including alterations in gyrase genes, porins, regulatory systems, and efflux pump expression, were not evaluated. Similarly, additional components influencing genome evolution, such as defense systems, HGT predictions, and heavy metal resistance determinants, were beyond the scope of the present study. The geographic and lineage composition of the dataset represents an important limitation. ST621 accounted for 198 of the 301 MDR genomes (65.8%), while genomes from the United States (*n* = 256) and Brazil (*n* = 36) together comprised 97.0% of the dataset. Therefore, the observed resistome, virulome, mobilome, and pangenome profiles may partly reflect the predominance of ST621 and the geographic composition of the sampled genomes and should not be interpreted as broadly representative of the global MDR *P. aeruginosa* population. Furthermore, the genomic predictions were not supported by functional validation or transcriptomic analyses. Despite these limitations, the large and diverse genome collection provides a comprehensive overview of the genomic landscape of clinically relevant *P. aeruginosa* isolates. Overall, our study highlights the complex interplay among population structure, accessory genome evolution, ARGs, VFs, and MGEs in shaping the emergence of MDR *P. aeruginosa*. These findings enhance the understanding of the genomic basis underlying adaptation and resistance in this clinically important pathogen and provide a valuable framework for future surveillance, molecular epidemiology, and development of strategies to combat antimicrobial resistance.

## 5 Conclusion

This study provides a comprehensive genomic characterization of 301 MDR *P. aeruginosa* genomes, integrating population structure, pangenome structure, resistome, virulome, and mobile genetic elements. The findings demonstrate a substantial burden of acquired antimicrobial resistance determinants, accompanied by diverse mobile genetic elements, including plasmids, integrons, insertion sequences, and prophages. ARG–MGE association analysis further identified resistance determinants within plasmid-associated contigs and recurrent genomic contexts, including conserved arrangements involving predominant acquired ARGs, highlighting the potential contribution of mobile and genomic contexts to the persistence and dissemination of antimicrobial resistance. The identification of internationally recognized high-risk clones within the dataset further emphasizes the circulation of successful resistant lineages. Overall, this study provides insights into the genomic landscape and mobility-associated features of antimicrobial resistance in MDR *P. aeruginosa* and establishes a valuable resource for genomic surveillance and molecular epidemiology. Continued whole-genome sequencing-based surveillance, particularly with integration of ARG, MGE, and genomic-context information, will be important for monitoring the emergence and dissemination of resistance and supporting infection-control and antimicrobial-stewardship strategies.

## Data Availability

The original contributions presented in the study are included in the article/Supplementary material, further inquiries can be directed to the corresponding author.

## Footnotes

1
https://pubmlst.org/organisms/pseudomonas-aeruginosa
