# Supplementary material for: Genomic insights into population structure, resistome, virulome, and mobilome of multidrug-resistant Pseudomonas aeruginosa
Source: Front Microbiol. 2026 Oct 22;17:1940877. doi: 10.3389/fmicb.2026.1940877 (PMC13736605; doi:10.3389/fmicb.2026.1940877)
Supplement: Supplementary file 1 [file Supplementary_file_1.docx]

**Supplementary Figure 1:** Geographic distribution and collection timelines of multidrug-resistant (MDR) isolates

**Supplementary Figure 2:** Abundance and family distribution of IS across the genomes
